# A case study of outsourced primary healthcare services in Sindh, Pakistan: is this a real reform?

**DOI:** 10.1186/1472-6963-14-277

**Published:** 2014-06-25

**Authors:** Sana Tanzil, Aysha Zahidie, Adeel Ahsan, Ambreen Kazi, Babar Tasneem Shaikh

**Affiliations:** 1 Department of Community Health Sciences, Aga Khan University, Stadium Road, 74500 Karachi, Pakistan

**Keywords:** Contracting, Primary health care, Basic health units, Pakistan

## Abstract

**Background:**

Since a decade, low and middle income countries have a rising trend of contracting their primary healthcare services to NGOs. In Pakistan, public sector often lacks capacity to effectively & equitably manage the healthcare services. It led the government to outsource the administration of primary health care services to a semi-autonomous government entity i.e. Peoples’ Primary Healthcare Initiative (PPHI). This small scale study has assessed the quality of healthcare services at the contracted Basic Health Units (BHUs) with the PPHI and compared it with those managed by the local district government in the province of Sindh.

**Methods:**

A cross-sectional mix methods survey was conducted in November 2011. Two BHUs of each type were selected from the districts Karachi and Thatta in Sindh province. BHUs were selected randomly and a purposive sampling technique was used to recruit the study participants at the two study sites. Focus group discussions were conducted with patients visiting the facility while in-depth interviews were conducted with service providers. An observation based resource availability checklist was also administered.

**Results:**

There was a significant difference between the PPHI and the district government administered BHUs with regard to infrastructure, availability of essential medicines, basic medical appliances, mini-lab facilities and vehicles for referrals. These BHUs were found to have sufficient number of trained clinical staff and no punctuality and retention issues whatsoever. The district government administered BHUs presented a dismal picture in all the aspects.

**Conclusion:**

Out-sourcing of primary healthcare facilities has resulted in significantly improved certain aspects quality and responsiveness of primary healthcare services. This strategy is likely to achieve an efficient and perhaps an equitable healthcare delivery in low and middle income countries where governments have limited capacity to manage healthcare services.

## Background

The evidence from low and middle income countries shows a considerable difference between quality of healthcare services delivered by public and private providers. This difference in quality of care is more relatively more obvious at the level of facility management [1]. A systematic review of private and public health care services in low and middle income countries suggest that private sector is performing better in term of drug availability and aspects of delivery of care, including responsiveness and is relatively more client orientated [2]. This lack of capacity within public sectors has resulted in an increased trend of contracting their primary health care services to non-government organizations, non-state actors or to autonomous entities within the government system. The literature however is still not able to conclude the effectiveness of outsourcing as a type of health system reform because the evidence from different parts of world shows variable outcomes [3]. The studies from Pakistan, Bangladesh, India and Cambodia indicate that non-governmental entities did better even when they had the same or fewer resources than the public institutions [1,4]. On the other hand, contracting out examples from Jordan, Lebanon and Tunisia, still fail to show expected results and success in providing an effective and equitable healthcare delivery even after years of a health reform. Many countries such as Afghanistan, Egypt and Islamic Republic of Iran, experience with outsourcing is comparatively new and hence it is not appropriate to make conclusions before providing the system enough time to show remarkable achievements [1,4,5].

In Pakistan, lack of government’s capacity to manage healthcare services and failure to ensure accessibility and equity have compelled the country to outsource the primary health care services mainly the Basic Health Units, and to hand over the management to the non-government or autonomous organizations. People’s Primary Healthcare Initiative (PPHI), formerly known as the Prime Minister Healthcare Initiative was started in October 2005 to contract out the first level healthcare facilities (FLCFs) in all four provinces to a semi-government body the Punjab Rural Support Program [6,7]. Successful experiences led the government to scale-up this contracting model for the rest of the FLCFs in other parts of the country. In Sindh, currently 23 districts are working under PPHI management and public feedback is reported to be fairly encouraging [7]. Nevertheless, the debate is still on regarding long term implications of this particular type of health reform. Evidences support the performance of PPHI BHUs but failed to show significant differences due to too early evaluations without providing sufficient times to show significant improvement in healthcare with equity and increased accessibility [8].

New evidences based on health systems research are required to assess the actual effect of such health reforms and comparing them to the health facilities still managed by the government itself. Such evidence will allude to the issues and challenges *en route* to develop a sustainable and responsive health system. Therefore for our purpose we identified a list of variables to assess the difference in quality of care between two facilities as average travel time to reach BHUs (in minutes), average number of patients attending BHUs, average waiting time at BHUs (in minutes) and others as listed in Table 1.

**Table 1:** Comparison of the characteristics of PPHI and DG administered basic health units

**Facility characteristics**	**PPHI administered primary health canter facility**^**1**^	**DG administered primary health canter facility**^**2**^
**Average travel time to reach BHUs (in minutes)**	30 (±12.4)	30.67 (±11.3)
**Average no. of patients attending BHUs**	250 (±70)	75 (±35)
**Average waiting time at BHUs (in minutes)**	35 (±8.16)	21.9 (±5.57)
**Average number of Patients per day attending OPD**	80	30
**Average number of healthcare staff present in OPD**	7	3
**Number of ambulance for referral of emergency cases**	1	0
**Number of dental surgeons available for dental procedures**	1	0
**Average number of Ultrasound machine**	1	0

^1^DG= District government.

^2^PPHI = Peoples’ Primary Healthcare Initiative.

### Objectives

The study objective was to assess the range and quality of healthcare services at the Basic Health Units (BHUs) in Sindh, administered by the district governments as compared to the Basic Health Units which are being contracted and now managed by the Peoples’ Primary Healthcare Initiative.

## Methods

This cross-sectional survey was conducted in November, 2011. We selected two districts of the province Sindh as our main study sites based on the difference in their administrative set up at the FLCF. The first one being the District Thatta, a rapidly growing district with heavy demands on the resources of the city, coupled with the general lack of interest on the part of the local administration and suffers from a severe lack of basic services which has resulted in contracting in to all its BHUs through PPHI (to rural health support program; a semi government entity) about 2 years back. Second one was the District Karachi, representing the settings where albums are still managed by the conventional set up of the District Health Department of Sindh government. The rapid expansion of the city has resulted in development of many peri-urban populations where BHUs are the only source for seeking health care.

Two BHUs were selected from each study site. Purposive sampling technique was used to select study participants and Denzin’s methodological triangulation technique was adapted to reduce bias and increase reliability of results, i.e. involving multiple investigators in data collection, using multiple techniques such as individual in-depth interviews, observations regarding available healthcare facilities and the focus group discussions.

The quality of healthcare services has various aspects. WHO has suggested six key dimensions of quality or attributes of the quality in health care. Among those attributes efficiency; delivering health care in a manner which maximizes resource use, accessibility*;* delivering health care that is timely, geographically reasonable, and patient-oriented; taking into account the preferences of service users are important attributes affecting patient satisfaction regarding quality of service delivery [9]. Hence we formulated questions focusing these important attributes of healthcare quality to fulfill study objectives. Some of these are:

1. What is the organization and structure of BHU?

2. What type of services and healthcare facilities are provided at BHUs?

3. What are some of major barriers faced in implementation of Primary Healthcare?

4. What are the patients perception regarding service quality and reasons for attending that particular healthcare facility?

5. What are the suggestions of health care providers and patients for improvement of the health care system?

Data collection tools were developed after a thorough literature review and nominal group discussion among the study group members. An open ended semi-structured questionnaire was then developed and reviewed by the investigators for its content validity using the Delphi technique. A checklist was also used for assessing the availability of essential service normally offered at the BHUs in Pakistan. For this study, ERC approval was taken from at each study site, all the available patients were invited to participate in the focus group discussions to know about their perspectives regarding the quality of care and range of services available at the FLCF. Individual in-depth interviews were conducted at each BHU with some of the service providing staff as doctors, lady health visitors, midwives, dispensers, EPI-staff and technicians. Informed consent was obtained from participants of focus group discussions and in-depth interviews. Data was collected by the trained post graduate Community Medicine residents. The details of study participants are provided in Table 2.

**Table 2:** Summarizing study population number, characteristics, data collection techniques

**Administrative authorities**	**Sampling technique**	**M:F**	**Assessment techniques and key persons interviewed**
^1^PPHI (A)	**Purposive sampling**	7:6	FGD with 2 dispensers, 2 LHVs, 2 female medical officer, 1 female Sonologist,
			FGD with 6 patients
			Individual interview with In-charge medical officer
			Individual interview from EPI-coordinator
PPHI (B)		8:5	FGD with 2 dispensers, 1 technician
			FGD with 2 midwives and 2 LHVs
			FGD with 4 patients
			Individual interview with female medical officer
			Individual interview with In-charge medical Officer
^2^DG (C)		7:11	FGD with 9 patients
			FGD with two LHVs and three LHWs
			Individual interview with 2 female medical officers
			Individual interview with 1 male medical officer
			individual interviews with 1 dispenser
DG (D)		4:5	FGD with 6 patients
			FGD with 2 midwives
			Individual interview with 1 Dispenser

^1^DG= District government.

^2^PPHI = People’s primary healthcare initiative.

### Ethical review

The study was ethically reviewed and approved by the ethical review committee the Community Medicine Residency Program of the Aga Khan University.

### Data analysis

Thematic Analysis applied for qualitative information by using software NVivo version II. Data was analyzed separately for two different categories i.e. BHUs contracted out to PPHI and the BHUs managed by the District Government. Themes were developed, discussed among researchers and validated as the data collection proceeded. Descriptive frequencies and the means were calculated for few quantitative variables using SPSS version19.

## Results

PPHI administered BHUs as compared to district administered BHUs showed significant differences in terms of infrastructure and the availability of essential services.

In PPHI managed BHUs, the quality of services was remarkably better with regard to the availability of qualified physician and other healthcare workers as compared to the BHUs managed by the district government itself. As far as patients’ satisfaction is concerned, most of the patients were satisfied with the services at PPHI managed BHUs. However, few sporadic complaints regarding long waiting times due to large number of patients were noted. Many patients also had to cover long distances to reach health facility. There were also complaints regarding long waiting time in PPHI administered BHUs (35 ± 8.16 minutes) as compared to district administered BHUs (21.9 ± 5.57 minutes) (Table 1). A female patient at PPHI managed BHU mentioned:

“We have to cover long distances by foot and it consumes lot of our time and then long waiting time is another issue for us, especially for housewives.”

(35 year old married lady, patient at PPHI managed BHU).

A wide range of medicines, basic medical appliances, mini-lab facilities and vehicles for referrals were available at PPHI managed BHUs. Moreover, Basic Emergency Obstetric and Neonatal Care (EmONC) services were also offered along with comprehensive antenatal care and immunization services in day time, up till 1 pm only. Dental care was provided by a qualified dentist and ultrasound facility was available twice a week on specified days (Table 3).

**Table 3:** Issues & hurdles faced in Implementation of PHC

**BHU as per its administrative authority**	**Observation regarding healthcare setting**
1. PPHI (A)	Well-equipped functioning Dental OPD, General and Obstetric OPD with ultrasound facility for Obstetric patients, Family planning facilities, Immunization facility including Rabies and Anti-snake venom globulins, broad range of essential medicines is available
2. PPHI (B)	Well-equipped functioning Dental OPD, General and Obstetric OPD with no ultrasound facility, Family planning facilities, Immunization facility including Rabies and Anti-snake venom globulins, all essential medicines are available
3. DG (C)	Delivery facility with very limited postpartum care. Very basic medical tools were found such as B.P apparatus and thermometer. No ultrasound facility or Mini lab services, limited essential medicines are available
4. DG( D)	No delivery facility, only antenatal care was provided. Very basic medical tools were found such as B.P apparatus and thermometer. No ultrasound facility or Mini lab services, limited essential medicines are available

Healthcare providers at the PPHI managed BHUs mentioned the lack of human resource as one of their main constraint in increasing services’ timings relative to the patient flow at each facility. Emergency services were offered only in day time, up till 1 pm only, which was identified as an important limitation and was the concern of many patients. A patient mentioned:

“We have to go miles away to avail medical care if one develops any emergency complain after1:00 pm, we wish, if the doctor can stay longer at this facility.”

(A 40 year old married male; patient at district government managed BHU).

The retention of healthcare providers was another challenge as many of them were not satisfied with their salaries. At both type of BHUs, all the services were provided free of cost, however, in the PPHI managed BHUs all the staff was punctual and qualified providing patient oriented care. Ambulance and trained driver were also available for in time referral of emergency cases to district hospital.

Unlike PPHI administered BHUs, the district government administered BHUs were found to have poor infrastructure, with limited resources. The rage of medicines available at district government administered BHUs is limited and medicines stocks in the district government administered BHUs were scarce; even the some essential medicines and basic medical equipment were not provided by the management (Table 4); shows an obvious comparison of medicines available at two distinctly managed BHUs.

**Table 4:** Comparison of medicines availability among PPHI and DG administered basic health units

**List of medications available in BHU pharmacy (comparison of PPHI with district government administered BHUs)**	**PPHI administered BHUs**	**District government administered BHUs**
Antibiotics	Ciprofloxacin, Amoxil, Gentamycin, Vibromycin, Metronidazole	Co-trimaxole, Metronidazole, Nalidixic Acid
Anti-hypertensive	Amplodipine, Atenolol	Linsinopril
Anti-diabetics	Glibaclinamide, Metformin	
Anti-pyretic	Paracetamol	Paracetamol
Analgesics	Paracetamol, Diclofenac Sodium, Ibuprofen, Ponstan-forte	Paracetamol, Ponstan-forte
Anti-fungal	Co-trimazole, Nystatin	Nystatin
Anti-septic	Pyodine, Tincture, Methylalcohal	Tincture, Methylalcohal
Cough syrup and anti-allergic	Chlophenaramine Syrup, Cetrizine tablet	Chlorpheramine tablet
Vitamin supplements	Folic Acid, Ferrous Sulphate, Vit-B Complex	Folic Acid, Ferrous sulphate
Ointments	Polyfax	Polyfax, Betnovate-N
Ant-acids	Ranitidine, Mg Sulphate, Omeprazole	Mg Sulphate
Parenteral fluids	Normal saline, Ringer lactate	Normal saline, Ringer lactate
Anti-malarial medications	Quinine, Primaquine	Quinine
Anti- tuberculosis	Myrin-p Forte	
Others	ORS, Premethrin Lotion, Traneximic Acid	ORS, Premethrin Lotion, Gention violet

Patients at district government administered BHUs were very much concerned regarding the shortage of free medicines. During the FGD, one of the female patients expressed her views and mentioned that:

“We are poor; that’s why we come here but it’s useless to come here if they cannot provide us free medicines for very minor illnesses.”

(27 years old, married woman; patient in district government managed BHU).

Staff punctuality was identified as one of the major issues in BHUs managed by the district government and particularly lack of physicians’ availability for consultation was one of the most reported complains by patients as well as dispensers working at the BHUs. Many of the visiting patients were unaware of the available resource physician assigned to their respective facility.

A patient’s expression about the availability of physician was that

“I trust more on the dispenser than the doctor who comes seldom and I am satisfied with the treatment provided by the dispenser”.

(37 years old female patient; in district government managed BHU)

Lack of appropriate antenatal care including the facility’s capacity to provide Emergency Obstetric and Neonatal care, lack of ultrasound facility, unavailability of minilab and dental care facilities were observed at the BHUs managed by the district government. Unavailability of ambulance facility in case of emergency or referral was another concern of the patients at the district government BHUs. Study observations showed that the PPHI managed BHUs were providing all the above mentioned essential healthcare services. On the other hand, the district government associated BHUs were found to have poor performance mainly owning to a weak infrastructure.

## Discussion

This study is an after only, cross-sectional comparison, and it is quite evident from the results that the primary healthcare services were better managed in the PPHI administered BHUs as compared to the district government administered BHUs. These results of our study concur with the findings of other studies conducted in Bangladesh and Cambodia where the contracted NGOs did much better than the public sector health centers [10,11]. Moreover those studies also indicated positive and significant effects of contracting health indicators of the communities served. Contracting of PHC services in Pakistan was first done in the district Rahim Yar Khan (RYK) where 104 BHUs were handed over to a nongovernmental organization and according to a third party evaluation utilization of BHUs, physical conditions, availability of drugs, staff punctuality improved significantly [12]. However, it is to be noted that in RYK, staff salaries were increased up to 150% while in PPHI administered BHUs low staff salary was one of the issues responsible for an insufficient human resource.

In our study too, the PPHI managed BHUs were found to have improved infrastructure, staff attendance and availability of essential medicines. Studies conducted in Guatemala and Romania reported a higher level of patient satisfaction under the contracted out health centers due to these factors [11,12]. Our finding is also consistent with third party evaluation report showing that availability of essential drugs was better in the PPHI administered BHUs, with 42.5% highly satisfactory or satisfactory against 13.9% in DDOH administered BHUs [12].

Patients complain about the long distance from their residence, for both PPHI as well as the district administered BHU. Studies conducted in different countries showed that one of the main aims of contracting strategies was to improve access to care. Our results do suggest that contracting in/out health care services does have an effect on access to care with respect to increase in the number of patients seeking care in PPHI administered BHUs as compared to district administered BHUs, however the average travel time was similar in BHUs administered by PPHI as well as of the district government. This could be a reason because of the limited number of facilities and the agreement with PPHI did not include the provision to increase the number of BHUs *per se*.

Complaints regarding long waiting time in PPHI administered BHUs (35 ± 8.16 minutes) as compared to district administered BHUs (21.9 ± 5.57 minutes) is a finding similar to the third party evaluation report in which 82.5% of PPHI BHU patients and 88.3% of DoH patients are seen within 30 minutes [12]. This could be attributed to the patients load which was approximately 3 times higher in PPHI administered BHUs than the district administered BHUs in our study. Similar increase in patient load was observed in study conducted in RYK in which there was fourfold increase in the service utilization immediately after contracting out strategy [13,14]. Similar results were also reported in studies conducted elsewhere [9,15]. According to healthcare providers, lack of human resource and retention of healthcare providers because of non-satisfactory salaries were the major challenges faced by PPHI administered BHUs. It was also highlighted in a study in which low salaries and poor human resource management was considered to be the main cause of lack of effectiveness of government programs as well as corruption [16]. In case of Pakistan it might be important to point out that PPHI cost the government the exact same amount as district managed BHUs. The point is that it’s not increased resources that explain the difference.

In BHUs administered by the district government, unavailability of physician was one of the major complaints which is also reported in another study conducted in the Attock district, Pakistan in which 20% of the respondent claimed that physicians were unavailable at BHUs and rural health centers. This is mainly because of inappropriate human resource management policies and regulations in Pakistan, which has eventually led to a complete lack of accountability and a laid back attitude among the physicians and other staff of primary health care, in particular [17,18].

Therefore, it is of utmost importance to enhance the capacity of the district governments to oversee and manage such contracts and ensure a transparent and competitive process of contracting basic health care services [19,20].

With the implications of contracting health care services to the semi government or private organizations, careful attention is also needed to safeguard ethical, methodological, accountability, sustainability and governance related issues in such reforms. Government must also define its role explicitly in designing, implementing, monitoring and regulating such contracts to ensure their sustainability.

It was a brief audit of BHUs administered by two different entities. The quality of services was assessed mainly using qualitative methodology and limited information was collected regarding quantitative variables i.e. average number of antenatal visits, average number of children vaccinated each day, staff salaries. Moreover limited numbers of in-depth interviews were conducted from doctors who might be the potential key informants due to work load at the time of interview. This study was conducted in a rural district of Sind Thatta currently facing sever health related challenges and poor health system performance. This might limit the application of findings in better performing districts of the country in terms of health system. However this study adds on our current information regarding performance of health care systems under two different entities.

## Conclusion

Out-sourcing of the primary healthcare facilities has resulted in significantly improved quality of healthcare services. This strategy seems to achieve an equitable healthcare delivery in the low and middle income countries where governments have a limited capacity to manage the healthcare services. Contracting may thus prove to be an effective way to rebuild and reorient the primary healthcare, hence contributing to the overall health system strengthening in the developing countries.

## Competing interests

The authors declare that they have no competing interests.

## Authors’ contributions

AZ and AK conceived the study and supervised the data collection. ST, AZ and AA performed the data collection and drafted and revised the manuscript. BTS conducted the critical review and added the intellectual content to the paper. All authors read and approved the final draft.

## Pre-publication history

The pre-publication history for this paper can be accessed here:

http://www.biomedcentral.com/1472-6963/14/277/prepub
